# Protocol for investigating the warping of spatial experience across the blind spot to contrast predictions of the Integrated Information Theory and Predictive Processing accounts of consciousness

**DOI:** 10.1371/journal.pone.0340593

**Published:** 2026-01-29

**Authors:** Clement Abbatecola, Bernard Marius ’t Hart, Belén M. Montabes de la Cruz, Lucy S. Petro, Cyriel M. A. Pennartz, Giulio Tononi, Karl J. Friston, Jakob Hohwy, Umberto Olcese, Melanie Boly, Andrew M. Haun, Srimant P. Tripathy, Patrick Cavanagh, Lars F. Muckli, TWCF-INTREPID consortium

**Affiliations:** 1 Centre for Cognitive Neuroimaging, School of Psychology and Neuroscience, College of Medical, Veterinary and Life Sciences, University of Glasgow, Glasgow, United Kingdom; 2 Imaging Centre of Excellence (ICE), College of Medical, Veterinary and Life Sciences, University of Glasgow, Glasgow, United Kingdom; 3 Centre for Vision Research, York University, Toronto, Ontario, Canada; 4 Swammerdam Institute for Life Sciences, University of Amsterdam, Amsterdam, the Netherlands; 5 Amsterdam Brain and Cognition, University of Amsterdam, Amsterdam, the Netherlands; 6 Department of Psychiatry, University of Wisconsin-Madison, Madison, Wisconsin, United States of America; 7 Wellcome Trust Centre for Neuroimaging, Institute of Neurology, University College London, London, United Kingdom; 8 Monash Centre for Consciousness and Contemplative Studies, Monash University, Melbourne, Australia; 9 School of Optometry & Visual Science, University of Bradford, Bradford, United Kingdom; 10 Department of Psychology, Glendon College, Toronto, Ontario, Canada; Museo Storico della Fisica e Centro Studi e Ricerche Enrico Fermi, ITALY

## Abstract

We investigate the subjective experience of space around the visual blind spot area, the cortical representation of which is missing feedforward connectivity from one eye. We performed these experiments as part of an adversarial collaboration to test contrasting theories of consciousness; Integrated Information Theory (IIT), Predictive Processing Active Inference (AI), and Predictive Processing Neurorepresentationalism (NREP) accounts. According to the Integrated Information Theory of consciousness, non-activatable retinotopic cortical regions, such as the blind spot region for the ipsilateral eye, create a different cause-effect structure and therefore should contribute differently to the perceived quality of space of activatable retinotopic regions. The two Predictive Processing accounts, in contrast, posit that internal models will accommodate structural deviations around the blind spot based on the available sensory evidence (particulars of this accommodation differ between the two accounts). We present a series of paradigms in which participants evaluate distances and areas that either include the blind spot or not (without stimulating it directly), as well as illusory motion that is either adjacent to the blind spot or not. We model psychometric functions relating perceived and objective space. These models vary in terms of bias and precision according to the experimental conditions (blind spot involved vs. not involved, ipsilateral vs. contralateral eye), making it possible to quantify the potential disruption of subjective spatial extendedness induced by the blind spot. We present simulated results for each experiment corresponding to the predictions of each account and conclude by discussing challenges and plans for dissemination.

## Introduction

At the cortical level, the region of the primary visual area that represents the physiological blind spot has structural deviations that can be studied during monocular viewing. Compared to other regions of our visual field which rely on feedforward information from the lateral geniculate nucleus (LGN), the cortical representation of the blind spot exploits its lateral and feedback cortical connectivity for filling-in processes ([1–4]). For this study protocol, three theories of consciousness, integrated information theory (IIT, [5]), and, in the context of predictive processing, active inference (AI, [6,7]) and neurorepresentationalism (NREP, [8,9]) make different predictions about the behavioural effects that the blind spot’s structural features, relative to non-blind spot regions, may have on the conscious perception of space. We present very compressed descriptions of the three theories and their predictions that are sufficient to motivate our experimental procedures. Further details on the three theories and the derivation of these predictions are available here: https://osf.io/35rhx.

IIT posits that when spatial judgements involve the blind spot, the difference in connectivity (i.e., absence of feedforward input) in that region would contribute to a different cause-effect structure relative to the one of a comparably eccentric region. According to IIT, the quality of spatial consciousness is determined by the specific composition of a cause-effect structure. Thus, the theory proposes that the perception of space should be altered when involving the blind spot region. By contrast, both AI and NREP are based on the assumption that perception relies on internal models that are optimised to reduce prediction errors. In the case of the blind spot, internal models should adapt to accommodate for the blind spot’s structural deviations. Specifically, NREP posits that lesions of portions of the visual field can have an effect on spatial estimates, but will be largely compensated for by the sensory evidence available from intact portions of the visual field. According to AI, the quality of spatial experience is determined by the cause-effect structure under a generative model apt for active vision. This model of projective geometry is not the geometry of anatomical projections. Thus, AI proposes that perceptual judgements should not be altered when involving the blind spot, other than possible changes in perceptual uncertainty, due to differences in sensory sampling (e.g., decreases in psychometric sensitivity). As a result, both predictive processing-based accounts (AI and NREP) propose that perceptual distortions should be either absent or small when involving the blind spot. These predictions were derived as part of a larger multi-experiment structured adversarial collaboration.

### Integrated information theory

Integrated information theory (IIT) claims that the quality of a conscious state is given by a maximally irreducible structure of the causes and effects of a system (e.g., of a neural nature), namely, its cause-effect power ([10]). In the case of visual space, the theory predicts that the experience of space is defined by the cause-effect power of a grid-like substrate and, due to its grid-like networks, proposes the retinotopic cortex as a plausible candidate ([5]). If changes in connectivity between neurons in the grid-like network of the retinotopic cortex affect the cause-effect power of the mechanisms it composes, the quality of spatial consciousness would also change: the maximally irreducible cause-effect structure would differ between differently-connected versions of the network. When applied to the blind spot, differences in connectivity between monocular and binocular cortex may lead to differences in the visual space within versus outside the blind spot region. This could be measurable when comparing spatial percepts that span or do not span the blind spot insofar as lateral connectivity in V1, that makes V1 a grid-like network, differs from binocular to monocular cortex in this region. Since there is *less visual cortex* corresponding to the blind spot ([11]), IIT can be taken to predict that it may feel like there is *less visual space* in the blind spot region.

### Predictive processing (AI, NREP)

Set within the general Predictive Processing framework, both Active Inference (AI) and Neurorepresentationalism (NREP) accounts are based on the assumption that perception relies on internal models that are optimised to reduce prediction errors, therefore working against perceptual distortions.

With respect to consciousness, AI claims that shifts in conscious perception require active inference, which refers to planning and decision-making in terms of inference of policies for action (such as decisions to shift attention, or engage in eye movement), where policies are inferred under the expectation they will reduce uncertainty about the hidden states of sensation ([6,7,12–14]). Uncertainty reduction equates to belief updating, which can be inferred through participants’ behaviour as a proxy of conscious perception. With respect to the physiological blind spot, AI proposes that the internal models compensate for structural deviations, arguing against perceptual distortions being associated with the physiological blind spot. However, given that internal models have noisier sampling in and around the blind spot region, AI predicts that judgements involving the blind spot should be more variable (i.e., no difference in terms of accuracy but less precision).

By contrast, NREP proposes that conscious perception arises from the interplay between low-level and high-level sensory inference, where low-level refers to inferential processing in neural ensembles and small networks, whereas ‘high-level’ involves inferences between submodalities (e.g., color, shape) and main modalities (e.g., vision and touch; [9,15]). When applied to spatial consciousness, NREP posits that the experience of extendedness arises from the integration of different spatial frames of reference (e.g., retinotopic, craniotopic, allocentric; [8]), relying on inferences made jointly across visual field portions, proprioceptive signals from eye and head movements, vestibular changes, etc. This integrative processing not only takes place in lower visual cortical areas (e.g., V1), but also in higher associative areas, such as parietal and middle temporal cortex ([16,17], cf. [18]). With respect to the blind spot, NREP predicts that contextual information derived from cortical regions representing intact portions of the visual field should enable the subject to roughly create the same global predictions about the causes of sensory stimulation as those derived from undistorted connectivity maps. Following this, NREP predicts there will be no major spatial disruption if spatial inference mechanisms can sufficiently rely on the intact portions of the visual field (i.e., portions around the blind spot) to produce accurate spatial parameter predictions (e.g., on distance). However, since it is possible that neural mechanisms infer spatial parameters using the through-blind spot information as well as around-blind spot trajectories, a modest decrease in spatial bias may be observed (‘modest’ understood as reaching a modestly significant p-value according to classical frequentist statistics).

### Current study

We describe a set of experiments that were developed based on predictions made by the three theories of consciousness that are tested in this adversarial collaboration (AI, IIT, and NREP). Our experiments form one protocol paper, which will be published alongside three others from the same adversarial collaboration (see https://osf.io/35rhx for all preregistered designs). Perceptual distortions across the blind spot have been tested before ([19,20]). The hypothesis that was tested in these papers was whether the missing neuronal representation is ‘stitched together at the level of retina’ or ‘cortex’ which would lead to substantial perceptual shrinkage around the blind spot. [19,20]) concluded that the distortions they observed were not big enough to favour the hypotheses of retinal or cortical stitching of retinal representations, but, instead, favoured a compensatory mechanism hypothesis, with little, or no, spatial distortion. In contrast, our aim is to assess whether the existence of the cortical blind spot affects the conscious experience of spatial extendedness for stimulation that does not stimulate the boundaries of the blind spot. For this purpose, it is essential that our procedures avoid effects of perceptual filling in processes as much as possible.

We propose three experiments that rely on estimating distance (Experiment 1), area size (Experiment 2) and motion curvature (Experiment 3) at locations that are near to the blind spot area (>1 degree visual angle – DVA – away) or away from the blind spot area for either the ipsilateral or contralateral eye through dichoptic presentation. The comparison between stimulus locations, and stimulated eye will allow us to assess the effects of the non-stimulated blind spot region on our measurements. All experiments will include eye tracking to ensure that our data are not influenced by stimuli triggering the boundaries of the blind spot as represented by receptive fields in the visual cortex (see [21–23]).

## Materials and methods

### Aim

We will explore the different theoretical accounts using three tasks quantifying different types of spatial perception in relation to the blind spot in healthy humans. In the distance task, participants will judge the distance of pairs of dots that either span or do not span the blind spot. In the area size task, participants will judge the apparent size of circles that either surround or do not surround the blind spot. In the motion task, participants will judge the apparent curvature of motion paths that either neighbour or do not neighbour the blind spot. Each task will be performed in a standard trial-based format, allowing us to measure psychometric functions relating stimulus parameters to perceptual judgments. From these functions, we can extract measures of judgment bias and precision (i.e., slope). In all three tasks, we will achieve stimulation around the perceptual blind spot by presenting stimuli dichoptically (see below).

### Status and timeline

In the latter part of 2020, two TWCF workshops were hosted online by one of the coauthors, Prof. Cyriel Pennartz, resulting in, (1) the formation of the TWCF Intrepid consortium, (2) the selection and design of four experimental paradigms capable of arbitrating amongst hypotheses derived from IIT, NREP, and AI-C. Out of these paradigms, we present the experimental protocols for ‘Experiment 2a’ (see https://osf.io/35rhx for all preregistered designs). From this, the consortium initially proposed a successful pilot grant, testing the viability and feasibility of the experiments in arbitrating between theories. After a successful piloting phase, a full grant application was submitted that was successfully awarded following peer-review. In mid 2021 (before the full grant application was submitted), the experiment team at University of Glasgow convened a meeting agreeing with Prof. Patrick Cavanagh from York University, Toronto and Dr. Srimant Tripathy from the University of Bradford, Bradford (UK) to form the replication lab. In late 2021, following the success of the TWCF pilot grant, the Glasgow Lab ran piloting for proof-of-principle for the eye-tracking and behavioural components of the three experimental tasks (pilot start = 03/11/2021, end 15/01/2024). This design was then pre-registered on OSF (https://osf.io/35rhx). Participants provided informed written consent for all pilot experiments. Currently, piloting and paradigm development have been completed for our three experimental tasks and data collection is underway. Recruitment has not been completed. We estimate completion of A) participant recruitment and B) data collection by the end of 2025. We also expect C) results by the end of 2025.

Based on this process, the experiments described herein form one of four protocols to be published, each describing experiments derived from predictions of the three theories being tested (AI-C, IIT, and NREP). Moving forward, consistent with the principles of co-design, all amendments to the experiment protocol following this publication will be addressed by agreement between the teams at both Glasgow and Toronto data collection sites, and the three Intrepid consortium theory leads (Karl Friston, Cyriel Pennartz and Giulio Tononi). Each amendment will be documented in experimental reports in OSF (https://osf.io/35rhx) identifying the specific change and rationale. A set of general analysis principles will be agreed upon prior to the commencement of the study. This process will be overseen by the Data Analysis and Replication Committee (DARC) of the Intrepid Consortium.

### Participants

We will conduct experiments in parallel at the School of Psychology and Neuroscience, University of Glasgow (Glasgow, Scotland) and at York University (Toronto, Canada). We will share programming scripts and methodology between sites.

In these experiments, we will test neurotypical subjects with normal or corrected to normal vision. We will only include participants with corrected to normal vision in cases where calibration works reliably (correction lenses sometimes reflect infra-red light, which can disturb eye tracking).

We will recruit participants through each site’s internal recruitment system. Participants give written consent. Participants in Glasgow will be reimbursed for their time at a rate of £7/h; participants in York will be reimbursed at a comparable rate in CA$20 or course credit. Each site’s human research ethics committees have granted ethical approval for this project (College of Science and Engineering Ethics Committee, University of Glasgow – Application Number: 300200313; Toronto – York University’s Human Participants Review Committee, Certificate e2019-194).

We will assess the presence of space distortions on perceived distance, area size and motion trajectories in areas neighbouring participants’ blind spot region. The use of small dot stimuli to study spatial vision near the blind spot has precedent (e.g., [20]). We hypothesise that effects might be small, so we will use a larger sample size (32 participants per experiment) than typical studies of perception across the blind spot (e.g., [3,20,24,25]).

### Apparatus

Stimuli will be composed of simple geometric shapes presented dichoptically to the ipsilateral eye (either across the blind spot or across a region neighbouring the blind spot) or to the contralateral eye (at the same locations). We will use PsychoPy ([26]) to code the protocols and to control the stimulation. Experimental scripts will be shared between the University of Glasgow and the replication lab at York University.

**Fig 1:**
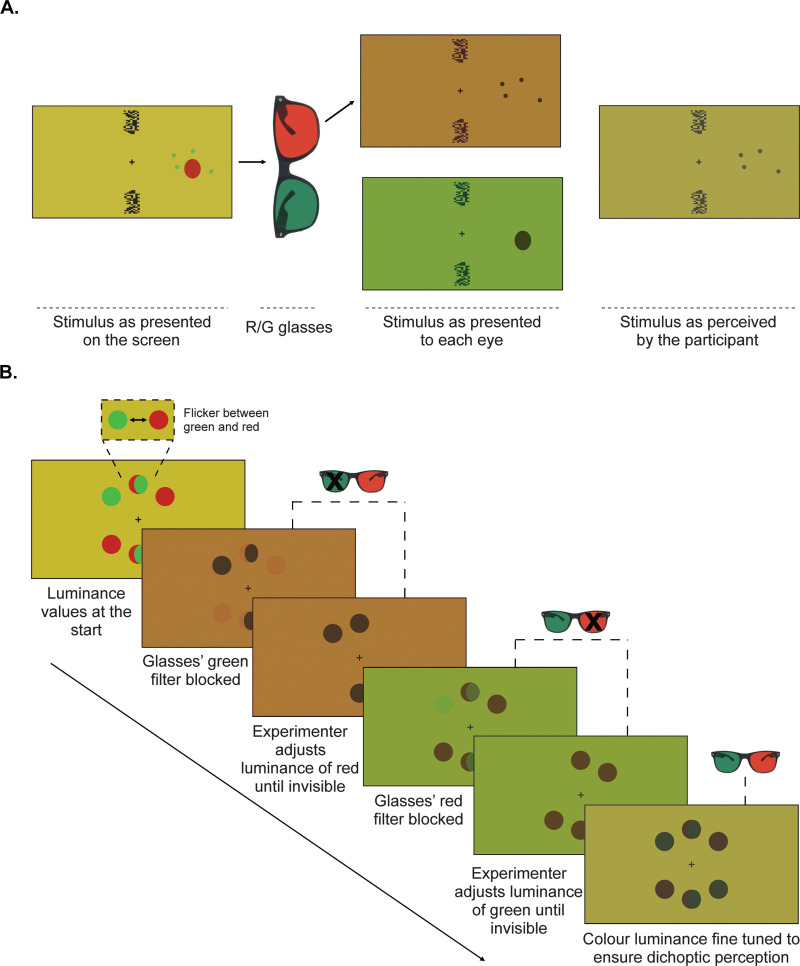
Dichoptic stimulation and colour calibration: A. Illustration of a given trial in which dot stimuli are presented to the contralateral eye (the left eye in this example). Through the use of the coloured glasses, the green coloured stimuli are visible to the contralateral eye alone, which is red filtered. By contrast, the blind spot patch is presented to the ipsilateral eye alone, which is green filtered. The dichoptic stimuli are experienced by the participant as if they were presented to both eyes. The blind spot mask is not visible to the participant (because of the ipsilateral blind spot, assuming correct fixation). To facilitate fixation, fusion stimuli are presented above and below fixation cross to both eyes B. Illustration of the colour calibration paradigm, in which the participant is presented with six dots located radially above and below the fixation. The experimenter blocks each coloured lens at a time to adjust the luminance of red and green separately. The final values of red and green luminance are experienced dichoptically as dark colours while remaining invisible through the respective lens filter.

At the University of Glasgow, the experimental procedure will be run on a Windows11 computer with an Intel(R) CoreTM i7-12700 CPU processor, an NVIDIA GeForce RTX 3070 and a 27“ DELL at 1920x1080 and 120 Hz. Participants’ viewing distance will be fixed at 57 cm from the screen using a chin and forehead-rest. Experimental stimuli will be presented dichoptically to participants with the use of red/green coloured glasses (see Fig 1A). We will record participants’ eye movements using an Eyelink 1000 eye-tracker camera (SR Research; Ottawa, Ontario, Canada) placed in front of the participant, below the screen.

At York University, the experimental procedure will be run on a Linux computer with an Intel© Core™ i7-12700K, an NVIDIA GeForce RTX 3060 and a 27“ACER at 1920x1080 and 144 Hz. The gamma will be linearized by PsychoPy using a colour characterization of the screen. Participants’ position and glasses will be as above. We will record participants’ eye movements using a LiveTrack Lightning eye tracker, (Cambridge Research Systems, Rochester, UK) at 500 Hz that will be placed ~30 cm (adjusted to calibrate individual participants) in front of the participant, below the screen.

### Colour calibration

To ensure that each stimulus is experienced dichoptically, participants will first complete a colour calibration task while wearing the red/green glasses (Fig 1B). In the task, participants will be presented with six coloured circles located radially from fixation, above and below the horizontal meridian. All circles will be coloured using PsychoPy’s rgb colour space (P-RGB; see Peirce et al., 2022 for details). Above the horizontal meridian, the leftmost circle will start faint green (P-RGB: [−1,.5, −1]) and the rightmost circle will start faint red (P-RGB: [.5, −1, −1]). The central circle will flicker between green and red every 200ms to help control subjective equivalence between these colours for the participant. By contrast, below the horizontal meridian, the leftmost circle will start faint red (P-RGB: [.5, −1, −1]) and the rightmost circle will start faint green (P-RGB: [−1,.5, −1]). The central circle will flicker between green and red every 200ms. To render the circles invisible through each coloured lens, stimuli will be projected on a khaki-coloured background made out of a mix of red and green (P-RGB: [.5,.5, −1]). We will use this same-coloured background during the blind spot mapping and for the main tasks.

The experimenter will block one coloured filter at a time as they adjust the intensity of green (up arrow increases value of green, down arrow decreases value of green) or red (left arrow decreases value of red; right arrow increases value of red). The participant’s task will be to select colour values that are invisible monocularly through their respective lens (note that while fixating the dichoptic perception is of dark colours).

### Eye tracking

Before beginning each task, participants’ eye movements will be calibrated using either the five-point calibration procedure (centre, upper, lower, left, right) and 5-point validation procedure (centre, upper, lower, left, right) of Eyelink 1000 (SR Research), or an analogous method in the LiveTrack system. Eye tracking will be recorded throughout the tasks and will be used to validate participants’ fixation. Eye gaze position will be described as the average position of both eyes. Correct fixation will be described as eye gazes that are less than 2 DVA away from the fixation cross, allowing for 1 DVA inaccuracy from each eye-tracker and 1 DVA of people’s fixation inaccuracy.

In the main tasks, a trial will start after participants have fixated correctly for 200 ms (minimum pre-trial fixation window). In cases where the participant fails to fixate correctly for 3s (maximum pre-trial fixation window) an automatic recalibration will be triggered. During trials when the participant’s gaze leaves the permitted fixation window (i.e., 2 DVA from fixation, checked at every frame refresh), the trial will be automatically aborted. When this happens, the fixation cross will change from a “+” shape to an “#’ as feedback that a broken fixation was recorded. Participants will be instructed to press the up key whenever this happens to return to the pre-trial fixation. We will also be able to perform re-calibrations manually with a key press (‘r’) to ensure that the eye tracking parameters remain accurate, and as a break from the fixation task whenever participants express tiredness (e.g., difficulty in fusing the fixation cross).

### Blind spot mapping

Following the eye tracking calibrations, participants will fixate monocularly (wearing the coloured glasses) at a cross located at the centre of the screen. Fixations will be indicated by changing the orientation of the fixation cross from an upstanding plus sign (i.e., + = fixation; gaze position < 2 DVA away from centre cross) to a 45 degrees rotated plus-sign (i.e., x = no fixation; gaze position > 2 DVA away from centre cross). Participants will be instructed that the cross should remain as an upstanding plus (i.e., +) throughout the task.

**Fig 2:**
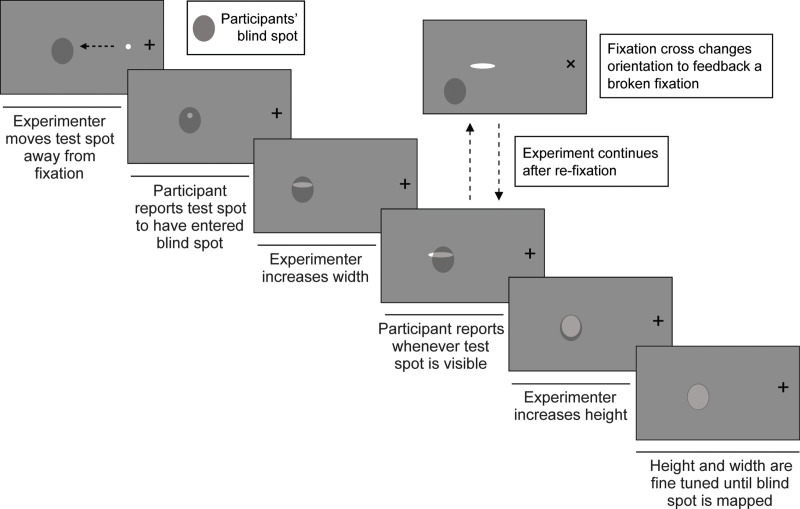
Blind spot mapping: The experimenter moves a test spot away from fixation along the horizontal axis until the participant reports the stimulus disappearing (i.e., to have entered their blind spot region). The experimenter then increases the width and height of the test spot until the participant’s blind spot is mapped (i.e., any further increase of the height or width of the stimulus would render it visible). The fixation cross changes colour whenever a broken fixation is recorded by the eye tracking equipment. The test spot should become visible when this happens (i.e., blind spot location shifts with gaze). The experiment continues once a fixation is recorded again. Note that while we represent the stimuli in black and white, the real task will use coloured stimuli and will be performed wearing the coloured glasses as shown in Fig 1A. This will allow us to present the stimuli monocularly to either eye depending on the blind spot mapping being performed.

Participants will see a small test spot (1 DVA of visual angle) adjacent to the fixation cross. The test spot’s colour (green or red, calibrated per participant) and original position (leftwards or rightwards from cross) will depend on the blind spot mapping that is being performed. As the participant fixates, the researcher will move the test spot along the horizontal axis (Y = 0) away from the fixation cross. Vertical adjustments will also be possible later in the procedure. The participant will indicate when the test spot disappears into their blind spot, at which point the researcher will incrementally enlarge its height (size increase along y axis) and width (size increase along x axis) in small steps until a section of the test spot becomes visible again. The test spot’s location and size will only be adjustable when the eye tracker records a central fixation (i.e., the test spot will be frozen on the screen whenever a missed fixation is recorded). The experimenter will be able to initiate a manual recalibration with a key press (r) in cases where the eye tracker records a missed fixation continuously. These recalibrations will also be employed to give the participant a break when needed. The experiment terminates once the maximum height and width has been established (i.e., any height or width increase or movement of the test spot in any direction would make the test spot visible. The test spot remains invisible as long as the participant fixates centrally). See Fig 2 for an illustration of the procedure.

### Main experimental design

Within each task, participants will complete a few training trials and then will engage in runs where stimulation is presented in the left hemifield (LH) and runs where it is presented in the right hemifield (RH). For LH runs, the left eye is ipsilateral and the right eye contralateral, while for RH runs the left eye is contralateral and the right eye ipsilateral. For each hemifield’s run, we will display a patch representing the previously mapped blind spot in the ipsilaterally-permissive colour (which is invisible to the contralateral eye as seen in Fig 1A). We will instruct participants that if the patch becomes visible at any point, or if part of the task-relevant stimulation disappears, it means that they lost fixation and should press a key (‘spacebar’) to abort the trial. Trials will also be aborted automatically whenever a lack of fixation is detected by the eye-tracker. Aborted trials will not be taken into account in data analysis or for staircase procedures (see below), they will be presented again later in the session until a valid response is recorded.

To ensure that dichoptic stimuli are presented at corresponding locations on the left and right retina (i.e., fused), we will present fusion stimuli at the centre-top and centre-bottom of the screen. The fusion stimuli will be vertical 15x3 DVA rectangular arrays (unit square = 0.5x0.5 DVA; distance task, motion curvature task) or horizontal 3x5 DVA rectangular arrays (unit square = 0.5x0.5 DVA; area task) composed of black- and background-coloured squares that will be randomised in arrangement between trials.

### Experiment 1: Distance task

**Fig 3:**
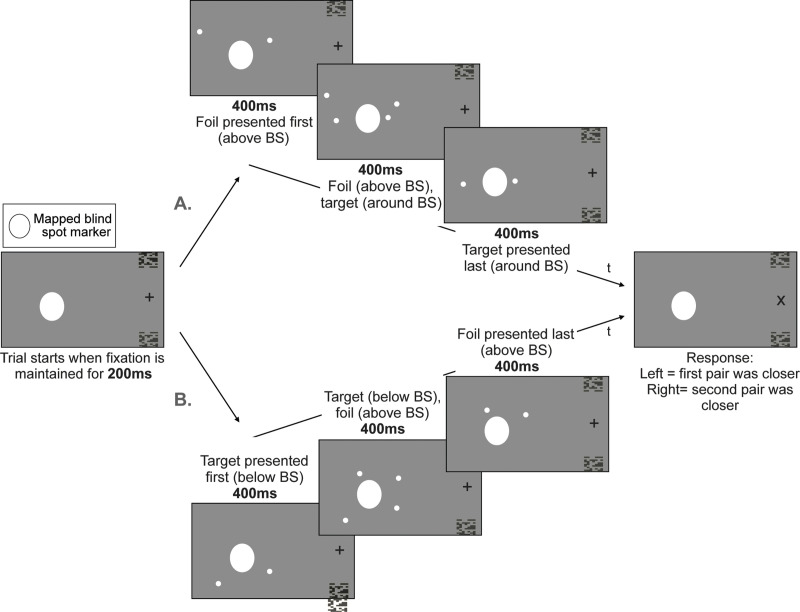
Distance task: We illustrate two given trials over time (t) in which A. the foil is presented first (above blind spot) for 400 ms, followed by a simultaneous presentation of the foil (above blind spot) and target (spanning blind spot) for 400ms and a presentation of the target alone (spanning blind spot) for 400ms and B. the target is presented first (below blind spot) for 400 ms, followed by a simultaneous presentation of the target (below blind spot) and foil (above blind spot) for 400ms and a presentation of the foil alone (above blind spot) for 400ms. All trials end with a fixation stimulus change from ‘+’ to ‘x’ which cues for a response to the task. Participants’ response is to state which pair of dots had a smaller separation, the one appearing first or second. Note that while we represent the stimuli in black and white, the real task will be coloured and will be performed with the coloured glasses as shown in Fig 1A. This will allow us to present the stimuli either to the contralateral eye or ipsilateral eye.

See Fig 3 for a schematic illustration of a single trial of this task. For each trial, we will show participants two pairs of dots sequentially, each for a duration of 0.8s with an overlap of 0.4s. Following the second pair’s disappearance, the fixation will change from a ‘+’ to an ‘x’ to prompt a response. Participants’ task will be to report whether the dots seemed closer to each other in the first or second appearing pair (2-alternative forced choice). Among the two pairs of dots, one, designated as the ‘target’, will always maintain the same distance, while the other, designated as the ‘foil’, will vary around the target distance (possible distance differences range from −3.5 to +3.5 DVA in steps of 0.5 DVA). The order of appearance of target and foil will be decided randomly at the start of each trial.

The target’s fixed dot-distance will be determined for each participant’s hemifield given their individually mapped blind spot area while accounting for dot size, padding to prevent stimuli from breaching the blind spot boundary (+2 DVA for each side of the blind spot – total of +4 DVA) and random jitter to prevent participants from using the retinal eccentricity of the more foveal dots to do the task (−1, −0.5, 0, + 0.5 or +1 DVA, same amount of jitter added to both dots). The distance difference between target and foil for a given trial will be determined by a staircase procedure: when participants select the target as being the smaller distance, the foil distance will decrease by 0.5 DVA for the subsequent trial in the staircase, and when participants select the foil it will increase by 0.5 DVA. For the dot pair spanning the blind spot, both dots are situated on a line including the fixation cross and the centre of the mapped blind spot location. For each trial, both dots are separated from the centre of the blind spot by half the target distance given the random jitter along the radial axis (Dot1 = Blind Spot Center – Target distance/2 + jitter; Dot2 = Blind Spot Center + Target distance/2 + jitter). In the case of the foil, the more central dot and the more peripheral dot of the pair are instead separated by the foil distance. To get the locations for dot pairs that do not span the blind spot, a polar angle representing the height of the blind spot plus 2° rotation is added (above location) or subtracted (below location) vertically to the centre of the blind spot.

We will show both target and foil for each trial either to the ipsilateral or contralateral eye. Crucially, while the foil dot pair will never span the blind spot (presented half the time above or below in a randomised order within a staircase), we will show the target dot pair either spanning the blind spot or not. For each of these combinations, we will define 2 staircases, one for which the foil distance starts as the smallest possible value (i.e., obviously smaller than the target distance) and one for which it starts at the largest possible value (i.e., obviously larger than the target distance). This yields a total of 8 staircases (2 eyes x 2 starting differences x 2 target pair locations) that will run interlaced to compose one run. One staircase procedure ends when it has lasted for at least 30 trials, and there have been at least 10 reversals (defined as a switch from choosing the foil to choosing the target or the opposite). At the start of each trial, one of the staircases that has not already finished will be randomly selected until all staircases are done. A full session of this task including calibration and two runs lasts approximately one hour and a half.

Using these data, we will model psychometric functions relating distance difference between target and foil to the proportion of times the target is chosen as being smaller in distance. These functions will be allowed to vary in terms of bias and precision according to the experimental conditions (around vs not around the blind spot, ipsilateral vs contralateral eye), making it possible to quantify potential disruption of distance perception induced by the blind spot.

### Experiment 2: Area task

**Fig 4:**
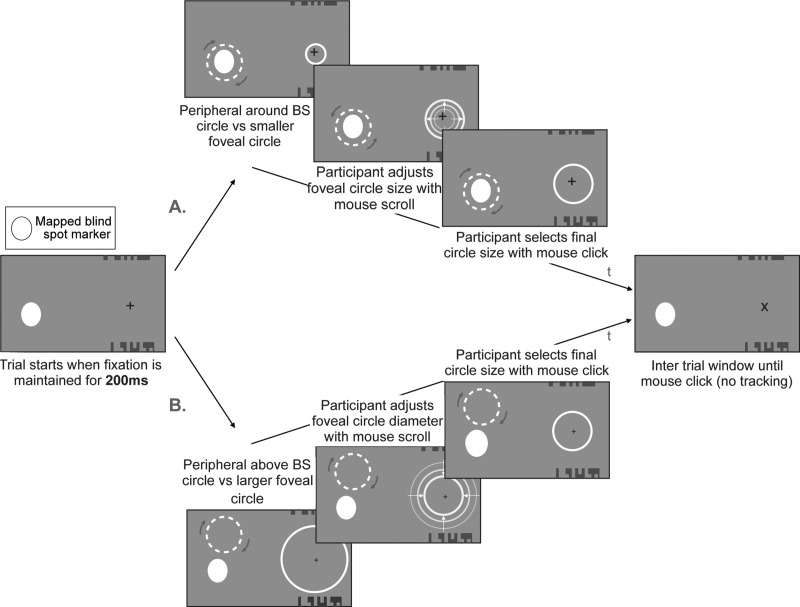
Area task: We illustrate two given trials over time (t) in which A. the foveal circle’s size starts smaller than the peripheral one and B. the foveal circle size starts larger than the peripheral one. Participants’ task is to adjust the foveal circle’s area to match the peripheral circle area. In all cases participants report their final size selection with a mouse click. The size of the fixation cross is randomised between trials to prevent local cues from biasing participants’ responses. Note that while we represent the stimuli in black and white here, the real task will involve coloured stimuli and will be performed with the coloured glasses as shown in Fig 1A. This will allow us to present the stimuli either to the contralateral eye or ipsilateral eye.

See Fig 4 for an illustration of a single trial of this task. For each trial, we will present participants with two circles, one foveally and one peripherally. The location of the foveal stimulus will be fixed around the fixation cross (“fixation” location) with some random jitter (−0.5 to 0.5 in steps of 0.05 DVA, with separate jitter applied to the x and y coordinates relative to the center of the stimulus). The peripheral stimulus will either surround the blind spot (“around blind spot” location) or be at an eccentrically comparable location (“above blind spot” location) with some random jitter (−0.5 to 0.5 in steps of 0.05 DVA, with separate jitter applied to both x and y coordinates relative to the center of the stimulus). Foveal and peripheral stimuli will differ in area. Participants’ task will be to adjust the area of the fixation stimulus to match that of the peripheral stimulus using their mouse. Participants will register the final size selection with a mouse click.

Peripheral circles will always be of fixed diameter, which will depend on each participant’s blind spot size and padding (peripheral diameter = participant’s blind spot size along longest dimension + 1.5 DVA of padding). The starting size of the foveal circle for a given trial will be randomly sampled from a distribution of smaller (5 values subtracted from the peripheral circle diameter in steps of peripheral circle diameter/10) and larger sizes (5 values added to the peripheral circle diameter in steps of peripheral circle diameter/10) with respect to the peripheral circle size. Fixation size will be adjusted by the participant by scrolling their mouse up (foveal circle diameter + peripheral circle diameter/10) or down (foveal circle diameter – peripheral circle diameter/10). Troxler’s fading occurs when an unchanging peripheral stimulus is experienced to disappear as a participant fixates centrally ([27,28]). To minimise this illusion, the peripheral circles will be dashed (12 dashes each spanning 15 degrees, and with a gap of 15 degrees between adjacent dashes) and will alternate between two orientations about its centre, by a polar angle of 15 deg, back and forth every 366ms.

For each trial, the circle stimuli (foveal and peripheral) will be presented dichoptically either to the contralateral or the ipsilateral eye, while the fixation cross and fusion stimuli will be presented to both eyes. To prevent participants from relying on local features to complete the task, the size of the fixation cross will be randomised across trials (sizes = 0.5 to 2.25 DVA in steps of 0.25 DVA) and jitter will be applied to both circles and to the fusion stimuli (jitter = 0.025 to −0.025 DVA in steps of 0.005 DVA, applied individually to x and y stimulus coordinates per trial). Foveal circles will always be presented surrounding the fixation cross (centrally plus positional jitter), while peripheral circles will be presented at a location that either surrounds the blind spot or is above the blind spot. For the peripheral location that surrounds the blind spot, circle stimuli will be situated centrally to the mapped blind spot location (plus positional jitter). To get the above location which does not surround the blind spot, a polar angle representing half the height of the blind spot plus the radius of the peripheral circle plus 2 DVA rotation of padding is added vertically to the centre of the blind spot.

Participants will complete a total of 50 adjustments (10 different start sizes: 5 larger than periphery, 5 smaller than periphery; each start size will be repeated 5 times) per eye and location (200 total adjustments) for each hemifield without a time constraint for responses. A full session of this task lasts approximately one hour.

Using this data, we will model the distribution of perceptual equivalence across trials, which will be allowed to vary in terms of bias and precision according to the experimental conditions (around vs not around the blind spot, ipsilateral vs contralateral eye), making it possible to quantify potential disruption of area perception induced by the blind spot.

### Experiment 3: Motion curvature task

**Fig 5:**
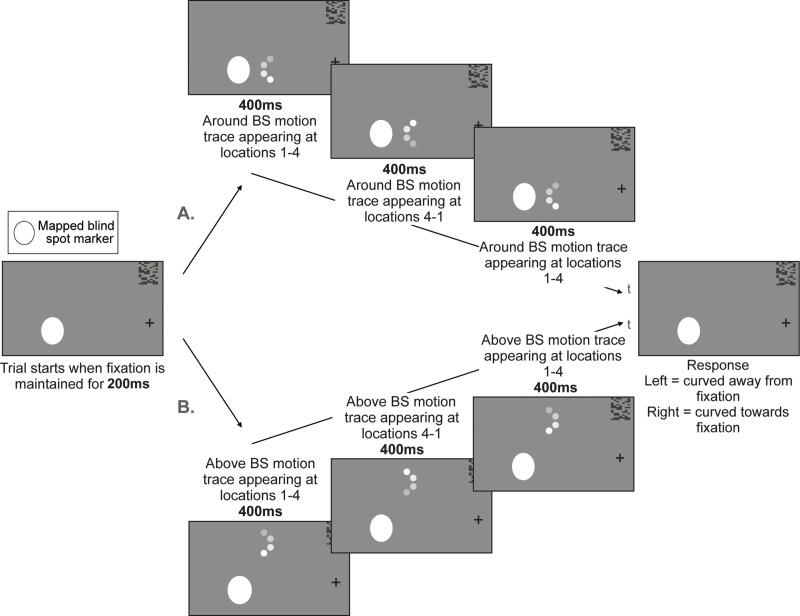
Motion curvature task: All trials begin with the presentation of the blind spot shape and central cross until the fixation is maintained for at least 200 ms. We illustrate two given trials over time (t) in which a motion trace is A. presented around blind spot, appearing at locations 1 (darkest gray circle), 2, 3 and 4 (white circle; 100ms/location). Following this, the same motion trace is presented appearing at locations 4 (darkest gray circle), 3, 2, 1 (white circle; 100ms/location) before appearing again at locations 1, 2, 3 and 4 (100ms/location) and B. is presented above blind spot appearing at locations 1, 2, 3 and 4 (100ms/location). Following this, the same motion trace is presented appearing at locations 4 (darkest gray circle), 3, 2, 1 (white circle; 100ms/location) before appearing again at locations 1 (darkest gray circle), 2, 3 and 4 (white circle; 100ms/location). All trials end with a fixation stimulus change from ‘+’ to ‘x’ which cues for a response to the task. Participants’ task is to state whether that trial’s motion path was curved towards the left (towards the blind spot in the left hemifield, towards fixation in the right hemifield) or the right (towards the fixation in the left hemifield, towards the blind spot in the right hemifield). Note that while we represent the stimuli in black and white, the real task will be coloured and will be performed with the coloured glasses as shown in Fig 1A. This will allow us to present the stimuli at different locations to either the contralateral eye or ipsilateral eye.

See Fig 5 for an illustration of a single trial of this task. For each trial, we will present participants with an apparent motion trace that will either appear neighbouring the innermost side of the blind spot (“adjacent to blind spot” location), or at an eccentrically comparable location (“above blind spot” location). Each apparent motion trace will be composed of 1 dot (diameter = 1.4 DVA) appearing briefly at 4 positions, all equidistant to their immediate neighbouring position. The trace’s second and third dot-positions will be located at a fixed distance from fixation (defined for each participant depending on blind spot coordinates and size). By contrast, the trace’s first and fourth dot-positions will be respectively displaced from the second and third dot-positions along a trajectory that will be curved towards or away from fixation (curvature ranges = −0.4 to 0.4 DVA^-1^ in steps of 0.05 DVA^-1^). This will create the perception of a curved motion (apparent motion curvature). The motion trace will be repeated so that the dot is going away from its starting position, returning to it and going away from it again before disappearing. Each motion trace will be followed by a change of fixation from a ‘+’ to an ‘x’ to prompt a response, and the task will be to determine whether the motion trace was curved towards or away from fixation.

The apparent motion curvature (i.e., the curvature by which the first and second dot-positions and the fourth and third dot-positions are displaced from one another) for a given trial will be determined by a staircase procedure: if the participant selects the motion trace as being curved towards fixation, the apparent curvature for the next trial in this staircase will be incremented in the opposite direction (less curved towards fixation/more curved away from it). Conversely, if the participant selects the motion trace as being curved away from fixation, the next apparent curvature will be incremented towards the fixation.

The exact locations of the motion traces (adjacent to and above blind spot) as well as the distance between the traces’ dot positions will be determined for each hemifield of each participant given their mapped blind spot area. For the adjacent to blind spot location, the second and third dot positions are above and below a point that connects the fixation cross with the centre of the mapped blind spot location. This point, on which the second and third dot positions are centred, is displaced from the centre of the blind spot by the participants’ blind spot size (blind spot width/2), the maximum width of the curved stimulus and padding (minimum = 0.5 DVA). The separation between the dots will be fixed at 2 DVA for all participants. The first and fourth dot positions will be displaced from the second and third by the curvature of a given trial (i.e., all dots will fall on a circle with radius = 1/curvature). All dots (1, 2, 3, 4) will be equidistant to their neighbours at all trials (distance1–2 = distance2–3 = distance3–4). The same procedure is used for the location above the blind spot except that a polar angle representing half the height of the blind spot plus 1.7° rotation of padding is added to the blind spot location.

Motion traces will be shown dichoptically to the ipsilateral or contralateral eye either adjacent to or above blind spot location. For each of these combinations, we will define 2 staircases, one for which the angle curvature starts as curved towards fixation (i.e., curvature = 0.4 for the right hemifield or curvature = −0.4 for the left hemifield) and one for which the angle curvature starts as curved away from fixation (i.e., curvature = −0.4 for the right hemifield or curvature = 0.4 for the left hemifield). This yields a total of 8 staircases that will run interlaced to compose one run. Each staircase procedure ends when it has lasted for at least 30 trials and 10 reversals.

Using this data, we will model psychometric functions relating apparent motion curvatures to the proportion of times they are chosen as being curved away from fixation. These functions will be allowed to vary in terms of bias and precision according to the experimental conditions (adjacent to vs above blind spot, ipsilateral vs contralateral eye), making it possible to quantify potential disruption of apparent motion perception induced by the blind spot.

### Data collection procedures

Participants will read and complete the information and consent forms using an anonymised subject ID. The participant will first complete the colour calibration and blind spot mapping tasks, which will follow the experimental procedure explained above. Then, the participant will take part in one of the three main experimental tasks (distance task or motion curvature task or area task). Each experimental task will follow the procedures outlined above. Since continuous fixation can be tiring, participants will be allowed to take a break whenever needed by asking the experimenter to call for a recalibration. Altogether, each experimental session is estimated to be completed in ~1h30 mins.

### Operational hypotheses

See Table 1 for a detailed description of each theories’ predictions. In this study protocol, participants will (i) compare the spatial separation between two dot-pairs (to compare distance), (ii) match the area of a circle relative to another (to estimate area size) and (iii) judge the curvature of apparent motion traces (to estimate apparent motion curvature) in trial-based psychophysics tasks. In all three paradigms we compare the accuracy (relating to bias) and precision (relating to slope) of responses to stimulation being presented near the blind spot location or at an eccentrically comparable location away from the blind spot (e.g., above), and for the ipsilateral and the contralateral eye.

**Table 1:** Table of experimental predictions.

Blind spot (ipsilateral) Predictions about the effect of stimulation around the blind spot on PSE (spatial bias)					
Theory	Decrease	No change	Increase	Confidence	Rationale
IIT	X			Medium	If there is less visual cortex in the blind spot area, it will feel like there’s less visual space in that region (for caveats cf. main text)
NREP	X	X		High	The distance between dots whose rectilinear connection goes through the blind spot, will be inferred by mechanisms using the through-blindspot line information (=incomplete) as well as using the around-blindspot trajectories. The overall estimate may be fully or incompletely corrected for the blind spot gap. Confidence is High that there is no change OR a modest decrease
AI		X		Medium	Perceptual inference reflects unbiased posterior estimate
Predictions about the effect of stimulation around the **blind spot** on **sensitivity (slope)**					
**Theory**	**Decrease**	**No change**	**Increase**	**Confidence**	**Rationale**
IIT	X			Medium	Extra-theoretical considerations suggest that variation in visual quality (btw. stimulated and filled-in qualities) may add to decision variability (a broader psychometric function)
NREP	X	X		Medium	The above ‘conflict’ between through-blindspot and around-blindspot trajectory information may decrease the sensitivity somewhat
AI	X			Medium	Precision estimates will be compromised by reduced information
**Blind spot (contralateral)** Predictions about the effect of stimulation around the **Corresponding Region of Contralateral Eye** on **PSE (spatial bias)**					
**Theory**	**Decrease**	**No change**	**Increase**	**Confidence**	**Rationale**
IIT	X			Low	Similar to ipsilateral rationale (visual space feels fused between L/R eyes – predictions differ to the extent that binoc. fusion fails)
NREP		X		Medium	Lack of information from contralateral field will not (or only slightly) affect distance estimation when dots are seen via ipsi (left) eye
AI		X		High	Perceptual inference reflects unbiased posterior estimate
Predictions about the effect of stimulation around the **Corresponding Region of Contralateral Eye** on **sensitivity (slope)**					
**Theory**	**Decrease**	**No change**	**Increase**	**Confidence**	**Rationale**
IIT		X		Medium	Extra-theoretical considerations suggest that similar visual quality may make for consistent stimulus comparisons
NREP		X		Medium	Lack of information in contralateral field will not (or only slightly) affect sensitivity of distance estimation when dots are seen via ipsi (left) eye
AI		X		Medium	Precision estimates will not be encumbered by any loss of information

**Fig 6:**
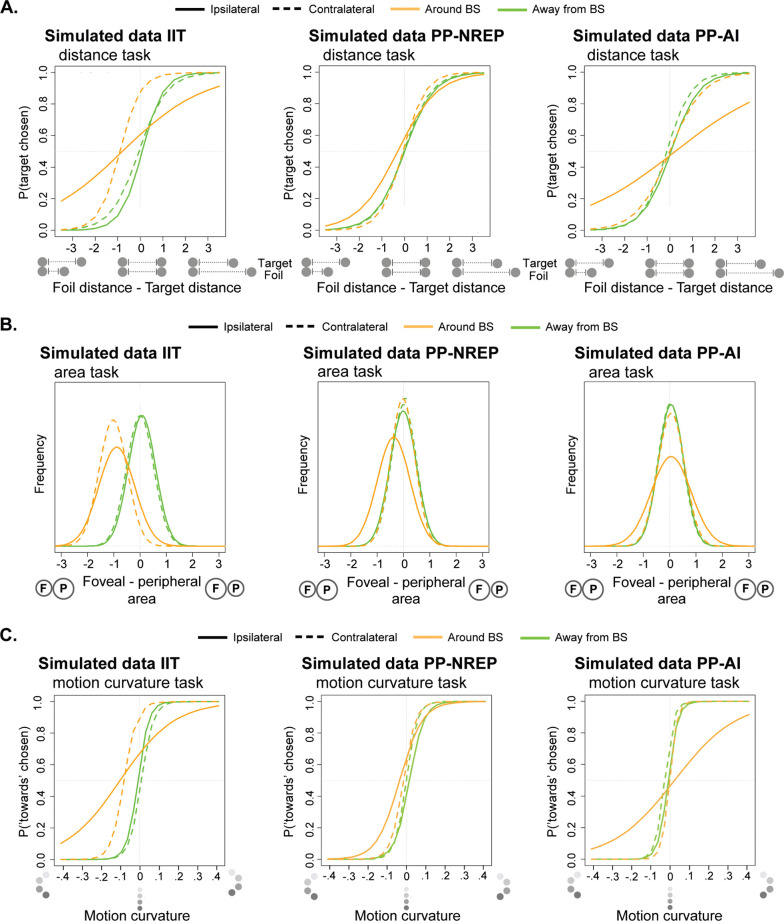
Simulated data: Simulated data of the three theories with respect to experiment 2.a Simulated data of IIT, NREP and AI predictions with respect to the A. distance estimation task, B. area size task and C. motion curvature task. See Table 1 for a detailed description of these predictions. A sample of the code used to simulate the predicted data represented in the figures is available at: https://github.com/ClementAbb/Intrepid_2a_staircase_simulation/tree/master.

Firstly, IIT proposed that differences in connectivity between monocular and binocular cortex may lead to a warping of visual space towards the blind spot region (i.e., a bias effect of the ipsilateral and contralateral eye for the blind spot location, Fig 6), causing space to be perceived as smaller when comparing spatial percepts that span – compared to those that do not span – the blind spot location. They further suggested that judgements could be more variable around the region of the perceptual blind spot (i.e., precision effect of the ipsilateral eye in the blind spot location, Fig 6). This second prediction resulted from considerations of Signal Detection Theory rather than IIT.

By contrast, NREP posited that the internal models should largely compensate for most of these structural deviations, arguing that differences in judgement accuracy and precision would be ‘small’ (modestly significant) or ‘non-existent’ between blind-spot and non-blind spot regions (i.e., small bias and precision effects for the ipsilateral eye in the blind spot location, Fig 6). The reasoning behind this prediction is that the spatial judgement will be mostly determined by inferences based on the available sensory evidence gathered from around the blind spot, whereas the (incomplete) through-blind spot sensory inputs (including the border regions) can nonetheless weigh into the decision.

Lastly, AI argued that the internal models should compensate for the entirety of the cortical differences between the blind spot and its neighbouring regions, proposing that judgement accuracy around the blind spot location should be unaffected (i.e., no bias effect for either eye). However, AI posited that since internal models may have noisier predictions around the blind spot, judgements could be more variable (i.e., less precise) when spanning the blind spot, but that judgement accuracy should be unaffected.

### Planned analyses and expected outcomes

Using the data from task 1, we will model the psychometric functions relating the distance difference between target and foil to the proportion of trials the target is chosen as being the smaller distance. Using the data from task 2, we will model the distribution of perceptual area size equivalence across trials. Using the data from task 3, we will model the psychometric functions relating apparent motion curvatures to the proportion of times they are chosen as being curved away from fixation. The resulting models, fitted using a probit link function, will be allowed to vary in terms of intercept (capturing bias effects) and slope (capturing precision effects) according to the experimental conditions (around vs not around the blind spot, ipsilateral vs contralateral eye). Thus, our modelling approach will quantify the potential disruption of spatial perception induced by the blind spot as bias and precision effects for either the ipsilateral or contralateral eye. This modelling approach will be implemented in two ways. We initially use a linear mixed effects model (lme4 package in Rstudio; see [29]), which includes random intercepts for participant and hemifield in addition to the fixed effects of our experimental conditions. This has allowed us to get a first estimate of the effect of eye (ipsilateral eye vs contralateral eye) or location (nearing vs away from blind spot) on the bias and precision of spatial perception while accounting for participant and hemifield response variability.

In the final stage, once data collection is completed for all experiments in the adversarial collaboration, we will use the Bayesian approach outlined by [30]. This framework was specifically developed for the purposes of comparing alternative theoretical hypotheses in the setting of adversarial collaborations. Briefly, this method involves specifying a set of generative models, where each model is equipped with a theory-specific prior derived from the predictions declared in this protocol and the OSF preregistration. These models are then fitted to the observed data using variational Bayesian techniques to obtain an estimate of the (log) model evidence (i.e., marginal likelihood or evidence lower bound), which quantifies the probability of the observed data under each model ([31]). These quantities can then be compared to assess how much evidence each theory has accrued for its predictions. This model comparison procedure is applicable in the context of our data as well as in the context of the wider adversarial project, making it particularly appropriate in our setting. Crucially, there will be no difference in the basic results for our experiments for the two approaches – by specifying neutral priors, this modelling approach will recover the same general bias and precision effects as described above. However, the Bayesian approach goes beyond these basic results, providing relative weights that will favour some theories over others.

### Data management and sharing plan

The management of intellectual property of study protocols in the adversarial collaboration, including the present one, will align with the European Commission and the guidelines of both institutions involved in the project, as will our policy for sharing behavioural, and eye tracking data. Specifically, we will adhere to the European Commission’s open access and data management policy and the European Commission’s Grants Policy on sharing of unique research resources.

All investigators agree to willingly share data and materials associated with this project so as to expedite future discoveries in consciousness and maximise the study’s impact. Following the acquisition of data, all data and any subsequent analysis will be uploaded to a shared server (EBRAINS, https://www.ebrains.eu/) available to all team members, as well as all members of the consortium and its associated committees. Over the life of the project, analysis and findings based on these data will be shared through multiple media, such as (including but not limited to) media appearance, lab meetings, conference talks and posters, and seminars (both at the host institutes and with the broader national and international scientific community).

In line with the principles of open science, de-identified data will be made available on an appropriate data repository. The specific items to be made publicly available as part of our resource sharing plan include:

1. All code used for stimulus creation, manipulation, and experiment presentation, as well as all code used to pre-process or analyse the data.
2. All (de-identified) eye-tracking, and behavioural data adhering to FAIR data principles. Despite creating no neuroimaging data in this project the larger TWCF ARC project will require the organisation of all raw data and derivatives to align with the Brain Imaging Data Structure BIDS format (http://bids.neuroimaging.io). All metadata will be made available.
3. Any peer-reviewed publication (the TWCF ARC project requires open access publications).

To allow the reuse of data—whilst also ensuring its integrity—downloading parties will be required to register before downloading any demographic, behavioural, eye tracking, or physiological data. A condition of the registration is that users agree: 1) not to distribute the data to third parties; 2) not to attempt to identify study participants; 3) to properly acknowledge the data resources. Data will be converted to shareable data formats (BIDS) and machine-readable annotations of the task and R/Matlab/Python scripts detailing the purpose of components will be included. There will be a GITHUB repository linked to the primary EBRAINS (or an equivalent alternative) repository that will be made available at the completion of the larger INTREPID project. We will review all data prior to upload to ensure they do not contain personal information or identifiable features. Data will be stripped of these details and will be General Data Protection Regulation (GDPR) compliant. We will share unprocessed (ASCII), minimally processed and final processed (CSV) data. All custom code and analysis pipelines will be shared. The experiment will be made available following publication via the OSF platform which links the user with all data being shared via EBRAINS (https://ebrains.eu/).

## Discussion

This study protocol forms one of four protocols (to be published separately) that were designed in an adversarial collaboration (Intrepid project) between information integration theory (IIT; [5]), and two branches of predictive processing, active inference (AI; [6,7]) and neurorepresentationalism (NREP; [8,9,15]). In the context of our study, the theories made differential predictions on the behavioural effects that the un-stimulated blind spot region may have on judgements of distance, area, and motion curvature. We will use these behavioural effects to assess the different theoretical predictions of the three theories (see Fig 6 for simulated data of each theory’s predictions). This will be done first through standard linear mixed-effects modelling, followed by a Bayesian analysis applied both within our protocol and across the broader collaborative framework, aiming to better understand the mechanisms behind the conscious experience of space.

Among the challenges that may affect our ability to arbitrate between the theoretical accounts and our observed data is, firstly, the fact that although the different theories predicted the direction of the effect on space, they did not specify the magnitude. For example, NREP argued that ‘small’ (down to ‘non-existent’) spatial disruptions could take place when judgements involved the un-stimulated blind spot region, where ‘small’ means a modest effect that is nonetheless significant (p < 0.05 according to frequentist statistics). Conversely, IIT proposed that space should be perceived as smaller around the blind spot region, but did not define how much of a disruption would be theoretically relevant.

A second outcome that could affect our arbitration is one in which we find an effect that was not predicted by any theory. For instance, we could find that space nearing the blind spot is perceived to be larger and not smaller (i.e., differing from IIT’s prediction), or that judgement precision around the blind spot location is affected for the contralateral eye and not the ipsilateral eye (i.e., differing from IIT’s and AI prediction). In this case, there may not be a straightforward explanation from any specific theory, but unexpected findings would still be valuable and could be used to further the frameworks’ future predictions. Another possibility is that different theories are supported by different experimental results (e.g., IIT supported by the distance task, NREP by the area task and AI by the motion curvature task), implying that the different theories are better fitted to explain different types of spatial judgements. In such scenarios, the results will be taken to refine or modify theories so that they may come to better explain non-confirmed results.

### The adversarial collaboration setting for this study

This study protocol forms part of a consortium project (Intrepid) engaged in adversarial collaboration among theories of consciousness, in this particular case IIT, NREP and AI (https://doi.org/10.54224/20646). In this consortium project, our experiment was specifically designed to test IIT’s prediction that differences in connectivity should lead to spatial distortions even in the absence of neural activity. As such, the results from this experiment are most informative with respect to IIT. Other experiments within the consortium were designed with different theoretical emphases and will therefore be most informative in those contexts.

The results for the basic predictions described above, as well as the results from the other experiments in this adversarial collaboration, will be analysed according to the Bayesian Adversarial Collaboration model comparison analysis scheme set out in [30], where all predictions from the theory leads are registered in protocols and at the OSF site. This Bayesian analysis will then be informative for further interpretation and discussion of the overall outcomes of the consortium’s findings. Other statistical tests (e.g., classical frequentist) may be conducted to further analyse the data, and analyses will be cross-validated by replication labs, primary labs and the teams of theory leads.

The proposed Bayesian Adversarial Collaboration model comparison analysis scheme is an evidence accumulation approach (see [30], for an in-depth discussion of its advantages over alternative approaches such as falsification). Our results should therefore be interpreted as contributing evidence toward the theory whose predictions best explain whether spatial distortions arise in the presence of the blind spot, with particular relevance for evaluating IIT’s claims.

### Dissemination plans

The outcomes of this experimental protocol and of the other experimental protocols in the Adversarial Collaboration will likely be published as a stand-alone article in the first instance or might be published as one combined paper. Each of these experiments and their components may or may not be presented through posters and talks at both international and regional conferences.

Following the completion and publication of the experiments described in each of the protocols published by the respective experimental groups, an integrative paper will be published that brings together and discusses the overall results in the context of the Adversarial Collaboration; a Bayesian analysis along the lines of [30] may follow or precede this paper.

Finally, the Intrepid Consortium plans to deliver public talks and a symposium to present and discuss the outcomes of the entire adversarial collaboration.

### Dealing with amendments

In the present experiment and the three other protocols that form part of the Intrepid consortium, amendments will be considered and agreed upon between the teams at both Glasgow and Toronto experiment sites, and the three Intrepid consortium theory leads (Karl Friston, Cyriel Pennartz, and Giulio Tononi). Upon the agreement of any reasonable amendments to the protocol, the amendment will be recorded and timestamped in the existing OSF preregistration documentation (https://osf.io/35rhx).

## Inclusivity in global research

Additional information regarding the ethical, cultural, and scientific considerations specific to inclusivity in global research is included in the Supporting Information (S1 Checklist).

## Supporting information

S1 Checklist CHECKLIST-Inclusivity-in-global-research-questionnaire. (DOCX)
